# The new Systematic Coronary Risk Evaluation (SCORE2 and SCORE2-OP) estimates the risk of arterial occlusive events in chronic myeloid leukemia patients treated with nilotinib or ponatinib

**DOI:** 10.1007/s00277-023-05556-0

**Published:** 2023-11-28

**Authors:** Olga Mulas, Elisabetta Abruzzese, Luigiana Luciano, Alessandra Iurlo, Immacolata Attolico, Fausto Castagnetti, Sara Galimberti, Massimiliano Bonifacio, Mario Annunziata, Antonella Gozzini, Ester Maria Orlandi, Fabio Stagno, Gianni Binotto, Patrizia Pregno, Claudio Fozza, Maurizio Loi, Malgorzata Monika Trawinska, Fiorenza De Gregorio, Daniele Cattaneo, Francesco Albano, Miriam Iezza, Claudia Baratè, Luigi Scaffidi, Chiara Elena, Valentina Giai, Emilia Scalzulli, Massimo Breccia, Giorgio La Nasa, Giovanni Caocci

**Affiliations:** 1 Department of Medical Sciences and Public Health, Hematology Unit, Businco Hospital, University of Cagliari, Cagliari, Italy; 2 Hematology Unit, Sant’Eugenio Hospital Tor Vergata University, Rome, Italy; 3 Hematology Unit “Federico II”, University of Naples, Naples, Italy; 4 Hematology Unit, Fondazione IRCCS Ca’ Granda Ospedale Maggiore Policlinico, Milan, Italy; 5 Hematology and Stem Cell Transplantation Unit, Azienda Ospedaliero-Universitaria-Consorziale (AOUC) - Policlinico, Bari, Italy; 6 Department of Experimental, Diagnostic and Specialty Medicine, S. Orsola-Malpighi Hospital, University of Bologna, Bologna, Italy; 7 Department of Clinical and Experimental Medicine, Section of Hematology, University of Pisa, Pisa, Italy; 8 Department of Medicine, Section of Hematology, University of Verona, Verona, Italy; 9 Hematology Unit, Cardarelli Hospital, Naples, Italy; 10 Hematology Unit, AOU Careggi, University of Florence, Florence, Italy; 11 Division of Hematology, Fondazione IRCCS Policlinico S. Matteo”, Pavia, Italy; 12 Hematology Unit, AOU Policlinico - V. Emanuele, Rodolico Hospital, Catania, Italy; 13 Hematology Unit, University of Padova, Padua, Italy; 14 Hematology Unit, Azienda Ospedaliero-Universitaria Città Della Salute E Della Scienza, Turin, Italy; 15 Department of Medical, Surgical and Experimental Sciences, University of Sassari, Sassari, Italy; 16 Hematology and Stem Cell Transplantation Unit, Department of Precision and Regenerative Medicine and Ionian Area (DiMePRe-J), University of Bari “Aldo Moro”, Bari, Italy; 17 Hematology, Department of Cellular Biotechnologies and Hematology, Sapienza University, Rome, Italy; 18 SC Ematologia E CTMO, Ospedale Businco, Dipartimento di Scienze Mediche e Sanità Pubblica, Università Di Cagliari, Via Jenner, Sn, 09124 Cagliari, Italy

**Keywords:** Chronic myeloid leukemia, Ponatinib, Nilotinib, Arterial occlusive event, Prophylaxis, SCORE2

## Abstract

Patients with chronic myeloid leukemia (CML) treated with nilotinib or ponatinib may experience arterial occlusive events (AOEs). It is currently recommended to thoroughly assess cardiovascular risk factors before treating CML. We identified 455 consecutive CML adult patients, 335 treated with nilotinib and 120 with ponatinib; 380 patients without previous cardiovascular diseases or diabetes were stratified according to the Systematic Coronary Risk Evaluation (SCORE2) and SCORE2-Older Persons (SCORE2-OP). This updated algorithm from the European Society of Cardiology (ESC) estimates a 10-year risk of fatal and non-fatal cardiovascular diseases. It is based on sex, age, smoking habits, systolic blood pressure, non-high-density lipoprotein cholesterol, and European geographical region of cardiovascular risk. The SCORE2/SCORE2-OP algorithm translated more patients (50.2%) to the high–very high cardiovascular risk category than the previous SCORE (25.3%). Patients with a high to very high SCORE2/SCORE2-OP risk showed a significantly higher incidence rate of AOEs (69.2% vs. 46.5%, *p* < 0.001). The older SCORE was less specific in estimating AOEs in patients classified as low-intermediate risk (69.8 vs. 54.2%). In multivariate analysis, no associations were found between AOEs and gender, age, and type or dose of tyrosine kinase inhibitor. Only the SCORE2/SCORE2-OP risk was confirmed as a significant predictive factor (*p* = 0.028; hazard ratio = 2.2; 95% confidence interval = 1.1–4.5). Patients with AOEs required, in most cases, imaging diagnostic tests, additional drugs, and sometimes invasive procedures, increasing access to visits and hospital management. This real-life study suggested that the SCORE2 and SCORE2-OP charts could help identify cardiovascular fragility in CML patients providing them with more attention and a proper TKI selection.

**Supplementary Information:**

The online version contains supplementary material available at 10.1007/s00277-023-05556-0.

## Introduction

Nowadays, the life expectancy of patients with chronic myeloid leukemia (CML) approaches the life expectancy of the general population [1]. Still, arterial occlusive adverse events (AOEs), which include coronary heart disease, stroke, and peripheral occlusive arterial diseases, represent serious complications of second- and third-generation tyrosine kinase inhibitors (2ndG/3rdG TKIs) [2]. Recent evidence has highlighted an increased AOE risk with 2ndG TKI nilotinib [3]. Ponatinib is a 3rdG TKI, active against native and mutated BCR::ABL1, indicated for the treatment of CML patients resistant and intolerant to 2ndG TKI and/or in the presence of T315I mutation [4]. Unfortunately, ponatinib treatment may induce cardiovascular adverse events, particularly AOEs. It is, therefore, essential to implement preventative measures and closely monitor the cardiovascular health of these patients [5]. Certain risk factors have been identified in developing AOEs, including basal cardiovascular risk factors and other factors such as a history of previous ischemic disease [6], dose intensity, and age at starting ponatinib [7]. Additionally, male sex, prior history of AOEs, and previous exposure to nilotinib can also contribute to developing AOEs [8].

In the high-income countries of Western Europe, cardiovascular diseases, which include AOEs, remain the most common fatal and non-fatal causes of morbidity and mortality. Therefore, identifying people at higher risk of AOEs should be crucial to adopt preventive actions [9]. In 2012, the European Society of Cardiology (ESC) identified a risk prediction algorithm known as the Systematic Coronary Risk Evaluation (SCORE) model, a 10-year risk estimation of fatal cardiovascular disease based on sex, age, smoking, systolic pressure, and total cholesterol level, to identify people at elevated risk [10]. The usefulness of the SCORE risk assessment to identify patients with increased risk of occurrence of AOEs during nilotinib or ponatinib treatment has been suggested [11–15]. Nevertheless, the SCORE presented some limitations, considering the age of people until 65 (most CML patients are older), including only fatal outcome prediction, and recruiting a cohort of control patients before 1986. Recently, the ESC has developed a new SCORE2, developed and validated on a large cohort of patients enrolled after 2000, aged 40–69 years [16], and a specific older person algorithm (SCORE2-OP), validated on patients over 70 years [17]. Differently from the older SCORE, which estimated a 10-year risk of fatal cardiovascular diseases, the SCORE2 and SCORE2-OP estimate a 10-year risk of fatal and non-fatal cardiovascular diseases and are not applicable to patients with a previous history of cardiovascular disease or diabetes because of the known high risk of cardiovascular complications in this cohort of patients. In addition, according to four geographical European risk regions, they are calibrated to the most contemporary and representative cardiovascular events. Interestingly, besides sex, age, smoking, systolic pressure, and European risk region, the chart displays the variable non-high-density lipoprotein cholesterol (non-HDL-C) instead of total cholesterol [16, 17].

Given the growing interest in the occurrence of cardiovascular events in CML as off-target effects in the long-term treatment with TKI and the need for algorithms on the proper stratification of cardiovascular risk in CML patients, we evaluated the estimating prediction role of the AOEs by the SCORE2/SCORE2-OP algorithm in a large real-life cohort of Italian patients treated with nilotinib or ponatinib.

## Methods

A group of adult patients with CML who received treatment with nilotinib and ponatinib between 2014 and 2018 was identified. These patients were treated in 16 different Italian medical centers. Information on baseline cardiovascular risk factors by reviewing medical charts was collected retrospectively. When assessing the SCORE2 and SCORE2-OP risk, patients were examined for age, gender, tobacco consumption, systolic pressure, non-HDL-C serum level at diagnosis, and European geographic region. Based on this evaluation, patients were categorized as either low to moderate or high to very high risk for cardiovascular issues. Some additional risk factors included having a body mass index > 24.5 kg/m^2^, mild or severe renal insufficiency, and dyslipidemia. During the evaluation, patients’ medical histories were checked for any pre-existing conditions and cardiovascular diseases, including cardiovascular events (CVEs) such as venous thrombosis, heart valve issues, arrhythmia, aortic aneurysms, high blood pressure, and AOEs such as myocardial infarction, angina, ischemic cerebrovascular events, and peripheral arterial diseases. A previous history of diabetes was also registered. It was noted whether antithrombotic prophylaxis was given before beginning CML treatment. Antithrombotic prophylaxis was defined as primary (based on baseline cardiovascular risk factors) and mainly represented by low-dose aspirin (100 mg/day). The prophylaxis was defined as secondary in the presence of a history of cardiovascular events and characterized by aspirin, other antiplatelet agents such as clopidogrel or ticlopidine, or anticoagulant therapy. We evaluated the cumulative incidence rate of AOEs after starting treatment with nilotinib or ponatinib and how hematologists and cardiologists handled them.

We compared stratified groups of patients using the log-rank test. To determine the incidence of AOEs, we studied the following variables: being male, age ≥ 60 years, receiving treatment with nilotinib versus ponatinib, and SCORE/SCORE2/SCORE2-OP risk impact. Multivariate analyses were performed using the Cox proportional hazards regression model. A *p*-value < 0.05 was considered statistically significant.

As per the guidelines set by the European Leukemia Net [18], the effectiveness of TKI treatment for CML patients was determined by their molecular response (MR). This was assessed by detecting the presence of BCR::ABL1 transcripts using a quantitative reverse transcription-polymerase chain reaction with a sensitivity level of 3 logs (MR3) or lower (MR4 and MR5) [19].

Data analysis was performed using the statistical package SPSS for Macintosh, Version 21, Chicago, IL. Patients signed informed consent under a protocol approved by the Registro Italiano LMC (Italian CML Registry), an initiative of the GIMEMA group (PROT. PG/2014/21960). The research was conducted under the aegis of the campus, an active research network of more than 50 Italian physicians involved in managing CML throughout the country, to investigate different aspects of the disease.

## Results

A total of 455 consecutive chronic CML patients (335 treated with nilotinib and 120 with ponatinib) were included in the study. Table 1 shows the patients’ characteristics. The median age at diagnosis was 50 years (range, 18–88 years). In 58% of patients, the Sokal score was intermediate to high. The median follow-up since CML diagnosis was 6 years (range, 1–20.8 years). A TKI treatment in the first line (nilotinib) was administered in 49% of patients, and most patients (51%) received a TKI treatment as a second line or subsequent lines of therapy (nilotinib or ponatinib) [20]. Among 335 patients treated with nilotinib, most received 600 mg/day (80%). Among 120 patients, ponatinib was administered at the following doses: 15 mg/day in 15% of patients, 30 mg/day in 35%, and 45 mg/day in 50%, respectively. Most patients treated with nilotinib (74.6%) showed a major molecular response (MR3), and 54.9% reached a deeper MR4 or MR5. Among patients treated with ponatinib, MR3 was found in 46.7% and MR4/MR5 in 21.7%.

**Table 1:** Characteristics of 455 CML patients treated with nilotinib or ponatinib

	Nilotinib, *N* = 335	Ponatinib, *N* = 120	Total, *N* = 455
Sex, *N* (%)			
Male	183 (55)	69 (58)	252 (55)
Female	152 (45)	51 (43)	203 (45)
Age at diagnosis, median years (range)	51 (20–88)	49 (18–70)	50 (18–88)
Median follow-up, median years (range)	6 (1–20.8)	7 (2–19)	6 (1–20.8)
Leukocyte × 10^3^/μL, mean value (range)	124 (7–898)	129 (10–1397)	87 (7–1397)
Hemoglobin g/dL, mean value (range)	12 (6–18)	11 (5–17)	12 (5–18)
Platelet × 10^3^/μL, mean value (range)	453 (31–1560)	383 (50–2290)	346 (31–2290)
BCR::ABL1 transcript type, *N* (%)			
p210	329 (98)	117 (97)	446 (98)
p190	5 (1)	0 (0)	5 (1.1)
p230	1 (0.3)	3 (3)	4 (1)
Splenomegaly, *N* (%)	157 (47)	68 (57)	225 (49)
Sokal score, *N* (%)			
Low	155 (46)	37 (31)	192 (42)
Intermediate	126 (38)	52 (43)	178 (39)
High	54 (16)	31 (26)	85 (19)
Line of treatment, *N* (%)			
First line	224 (27)	0 (0)	224 (49)
Second line	91 (27)	45 (38)	136 (30)
Third line	17 (5)	49 (41)	66 (15)
Fourth line	3 (1)	26 (22)	29 (6)
Nilotinib dose, *N* (%)			
450 mg/day	16 (5)		16 (3.5)
600 mg/day	268 (80)		268 (59)
800 mg/day	51 (15)		51 (11)
Ponatinib dose, *N* (%)			
45 mg/day		60 (50)	60 (13)
30 mg/day		42 (35)	42 (9)
15 mg/day		18 (15)	18 (4)
History of CVEs before TKI treatment, *N* (%)			
Myocardial infarction/angina	7 (2)	5 (4)	12 (3)
Arrhythmia	5 (1.3)	3 (3)	8 (2)
Other cardiac diseases	9 (3)	3 (3)	12 (3)
Peripheral arterial disease	3 (1)	2 (2)	5 (1.1)
Stroke	1 (0.3)	0 (0)	1 (0.3)
Hypertension	81 (24)	33 (28)	114 (25)
Peripheral venous disease	0 (0)	0 (0)	0 (0)
Diabetes	4 (1.3)	13 (11)	17 (3.7)
AOEs prophylaxis before TKI treatment, *N* (%)			
Primary prophylaxis*	44 (13)	19 (16)	63 (14)
Secondary prophylaxis**	11 (3)	6 (5)	17 (3.7)

*CVEs*, cardiovascular events; *AOEs*, arterial occlusive events

^*^Low-dose aspirin

^**^Low-dose aspirin, clopidogrel, and ticlopidine

At the diagnosis of CML, a positive history of cardiovascular disease was reported by 33.4% of patients, including hypertension, which was the most represented disease in the nilotinib (24%) and ponatinib (28%) cohorts (Table 1).

Primary prophylaxis of low-dose aspirin was ongoing in 13% and 16% of patients treated with nilotinib and ponatinib; secondary prophylaxis, including low-dose aspirin, clopidogrel, and ticlopidine, was adopted in 3% and 5% of the nilotinib and ponatinib cohorts.

At the time of diagnosis for CML, 23% of patients were found to have dyslipidemia. Specifically, 11% of patients had high (> 185 mg/dL) or very high (> 210 mg/dL) non-HDL-C serum levels, while the remaining patients had suboptimal levels (130–184 mg/dL). Of the patients with dyslipidemia, only 25% received antilipemic treatment. Overall, 75 patients with previous cardiovascular diseases or diabetes were excluded by the SCORE2/SCORE2-OP evaluation. Data on AOEs and CVEs occurring in these patients are shown in Supplemental Table 1, as well as the AOE cumulative incidence (Supplemental Fig. 1).

**Fig. 1:**
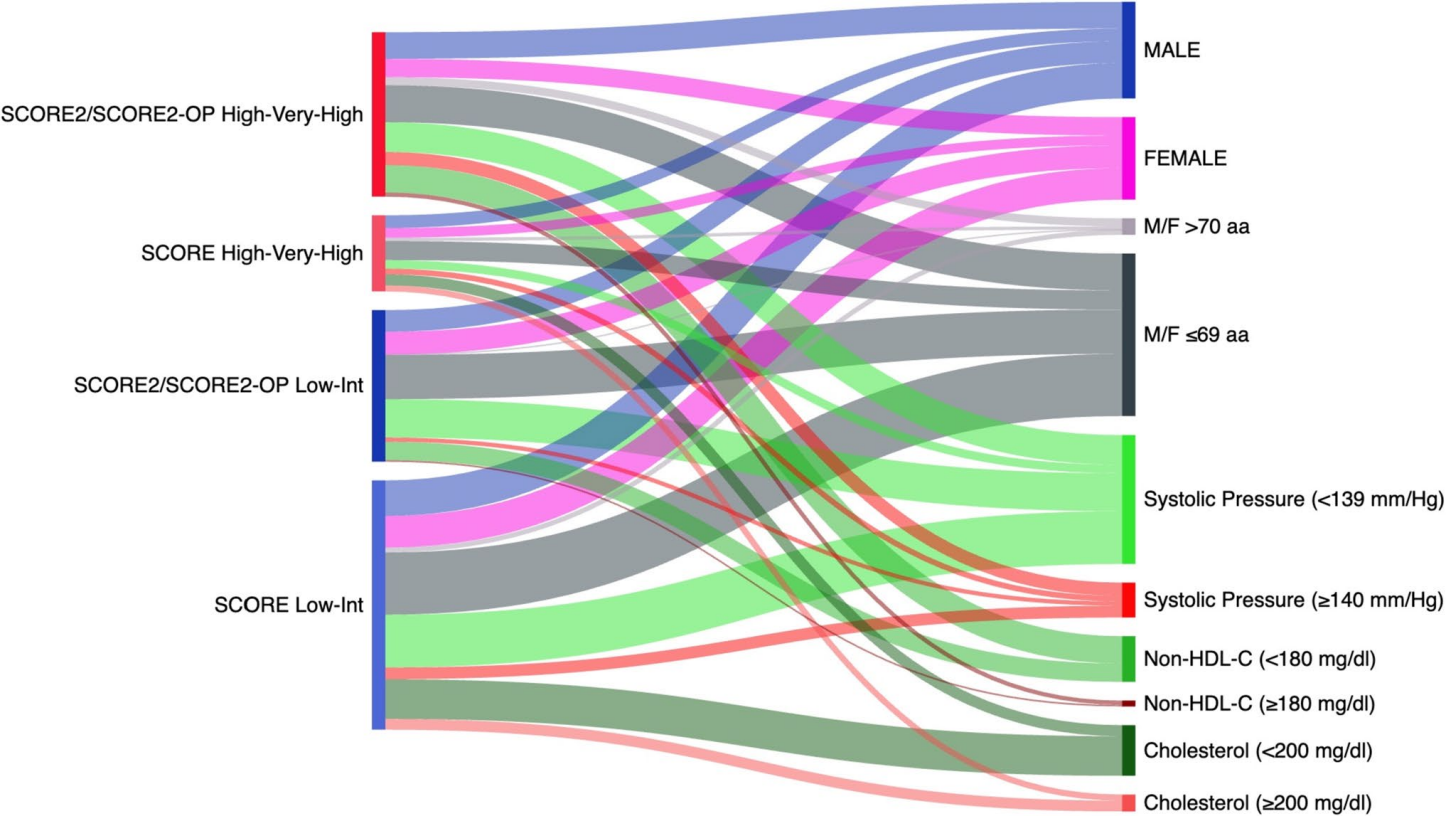
Sankey diagram showing the flow of 380 CML patients from the SCORE to the SCORE2/SCORE2-OP, following recalibration of the age- and sex-adjusted risk, based on the European area of cardiovascular risk and considering the variable non-HDL cholesterol instead of total cholesterol. The SCORE2/SCORE2-OP algorithm translated more patients (191 out of 380) to the high–very high cardiovascular risk category, compared to 96 out of 380 of the SCORE2

Figure 1 shows 380 patients classified with the cardiovascular risk SCORE and reclassified according to the SCORE2/SCORE2-OP risk chart evaluation. Applying the SCORE chart, 284 patients (74.7%) were classified as low to intermediate risk and 96 (25.3%) as high to very high risk. The SCORE2/SCORE2-OP algorithm translated more patients (191, 50.3%) to the high–very high cardiovascular risk category.

Overall, 33 AOEs were registered after the beginning of TKI treatment, 26 (9.1%) during nilotinib, and 7 (7.5%) during ponatinib (Table 2). Myocardial infarction, stroke, and peripheral arterial disease incidence were similar between the two groups. AOEs mainly occurred among patients treated with 600 mg of nilotinib and 45 mg of ponatinib. In the latter case, patients were often in the third or fourth line of treatment.

**Table 2:** Cardiovascular outcomes of 380 CML patients included in the SCORE/SCORE2/SCORE2-OP classification

	Nilotinib, *N* = 287	Ponatinib, *N* = 93	Total, *N* = 380
CVEs and AOEs following TKI, *N* (%)			
Number of CVEs	48 (16.7)	29 (31.2)	77 (20.3)
Arrhythmia	12 (4.2)	1 (1.1)	13 (3.4)
Hypertension	8 (2.8)	19 (20.4)	27 (7.1)
Other cardiac diseases	2 (0.7)	2 (2.2)	4 (1.1)
Number of AOEs	26 (9.1)	7 (7.5)	33 (8.7)
Myocardial infarction/angina	5 (1.7)	1 (1.1)	6 (1.6)
Peripheral arterial disease^±^	18 (6.3)	3 (3.2)	21 (5.5)
Stroke	3 (1)	3 (3.2)	6 (1.6)
Line of TKI at AOEs, *N* (%)			
First line	13 (4.5)	0 (0)	13 (3.4)
Second line	9 (3.1)	2 (2.2)	11 (2.9)
Third line	3 (1)	2 (2.2)	5 (1.1)
Fourth line	1 (0.3)	3 (3.2)	4 (1.1)
Dose of TKI at AOEs, *N* (%)			
45 mg/day		3 (3.2)	3 (0.8)
30 mg/day		3 (3.2)	3 (0.8)
15 mg/day		1 (1.1)	1 (0.3)
450 mg/day	1 (0.3)		1 (0.3)
600 mg/day	17 (5.9)		17 (4.5)
800 mg/day	8 (2.8)		8 (2.1)
Dose modification, *N* (%)			
Unchanged	7 (2.4)	0 (0)	7 (1.8)
Reduced	9 (3.1)	3 (3.2)	12 (3.2)
Interrupted	10 (3.5)	4 (4.3)	14 (3.7)
Therapies introduced, *N* (%)			
Coronary stents	2 (0.7)	0 (0)	2 (0.5)
PTA peripheral artery	1 (0.3)	1 (1.1)	2 (0.5)
Bypass	0 (0)	1 (1.1)	1 (0.3)
Aspirin	18 (6.3)	4 (4.3)	22 (5.8)
Anticoagulant	3 (1)	0 (0)	3 (0.8)
Other drugs®	14 (4.9)	2 (2.2)	16 (4.2)
No further action	1 (0.3)	0 (0)	1 (0.3)

*CVEs*, cardiovascular events; *AOEs*, arterial occlusive events; *PTA*, percutaneous transluminal angioplasty

^±^PAOD (peripheral arterial occlusive disease) and atheromatous carotid disease

®Diuretics, calcium channel blockers, ACE inhibitors, and beta blockers

**Fig. 2:**
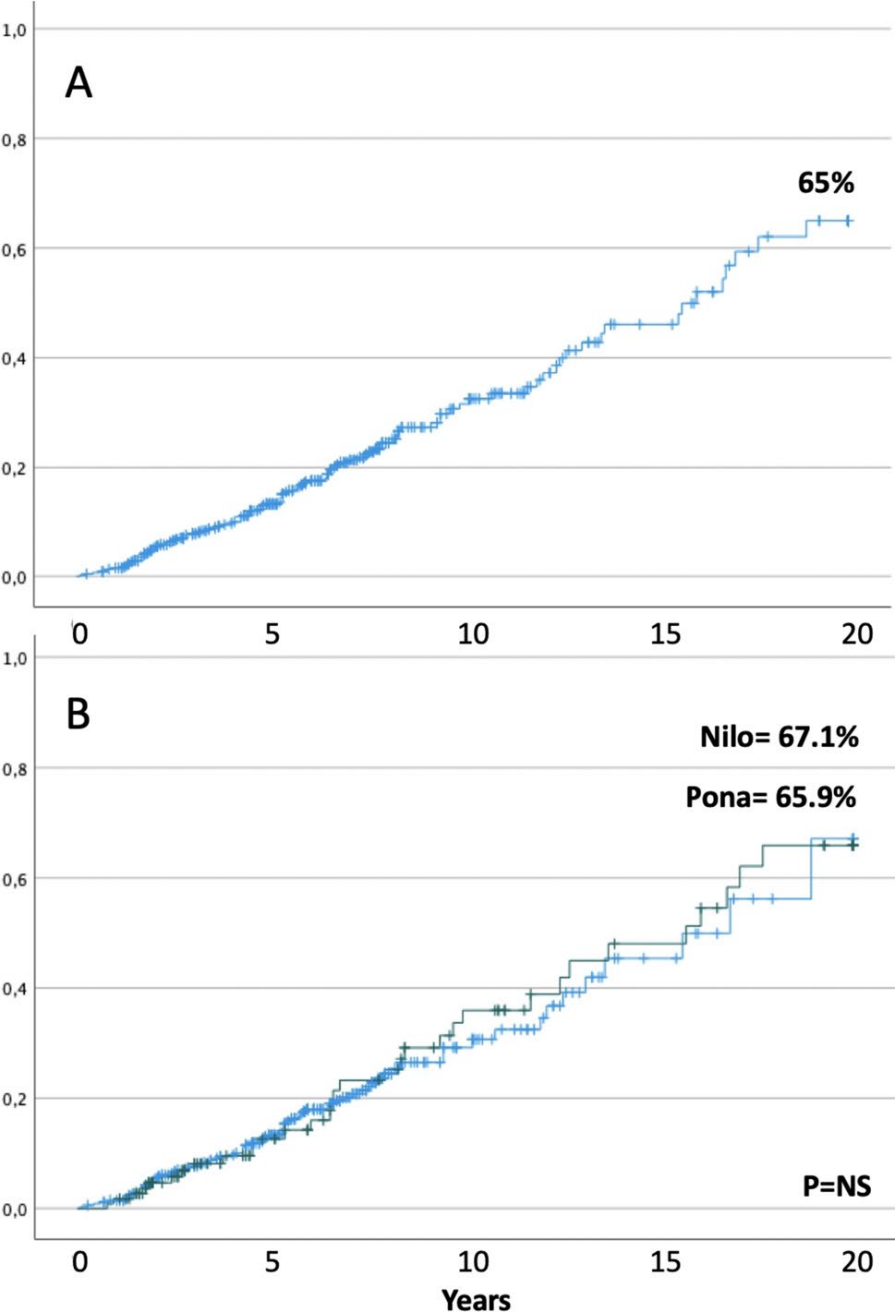
Cumulative incidence of arterial occlusive events in 455 CML patients (**A**) and according to therapy with nilotinib or ponatinib (**B**)

In the whole cohort of 455 patients, the 20-year cumulative incidence rate of AOEs was 65 ± 6.3 (mean 13.7 years; 95%CI = 12.8–14.6) (Fig. 2A). No difference was found between nilotinib and ponatinib treatments (67.1 ± 11.5 (mean 13.9 years; 95%CI = 12.7–15.1) vs. 65.9 ± 8.2 (mean 13.4 years; 95%CI = 11.8–15.1)) (Fig. 2B).

**Fig. 3:**
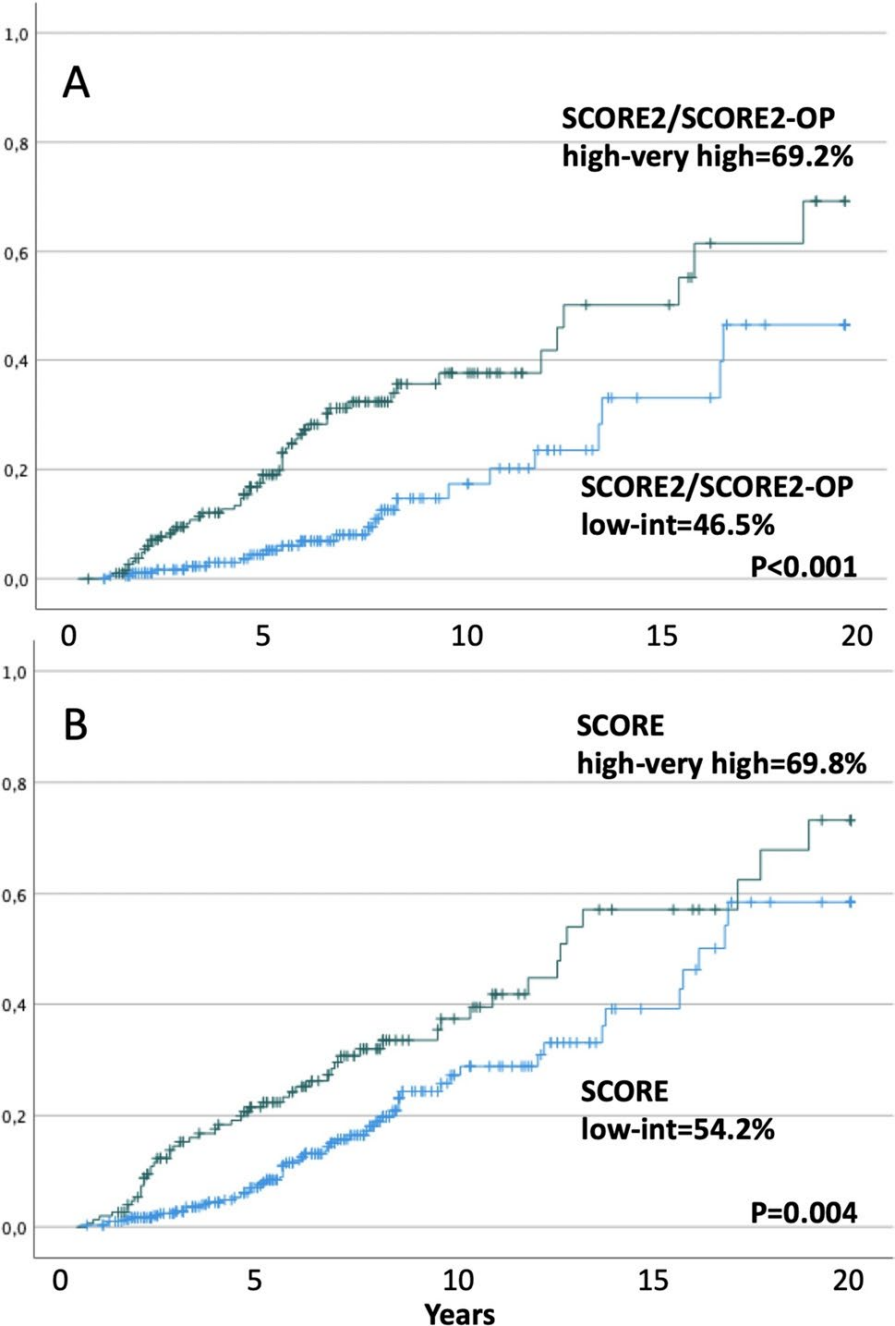
Cumulative incidence of arterial occlusive events in 380 CML patients treated with nilotinib or ponatinib, according to the SCORE2/SCORE2-OP algorithm, based on age, gender, tobacco consumption, systolic pressure, non-HDL-C serum level, and European region (**A**), and the older SCORE, based on sex, age, smoking habits, systolic blood pressure, and total cholesterol levels (**B**)

In the cohort of 380 patients included in the SCORE/SCORE2/SCORE2-OP evaluation, patients with a high to very high SCORE2/SCORE2-OP risk showed a significantly higher incidence of AOEs (69.2 ± 10.1 (mean 12.7 years; 95%CI = 11.3–14.3) vs. 46.5 ± 10.7 (mean 16.1 years; 95%CI = 14.6–17.5); *p* < 0.001) (Fig. 3A). The older SCORE equally predicted a significant cardiovascular event risk in higher risk patients. Nevertheless, estimating AOEs in those classified as low-intermediate was less specific than the SCORE2/SCORE2-OP (69.8 vs. 54.3%; *p* = 0.004) (Fig. 3B).

In multivariate analysis, no significant association was found between AOEs and gender, age, type of TKI, and the SCORE evaluation. The SCORE2/SCORE2-OP risk was significantly associated with the incidence of AOEs (*p* = 0.028; hazard ratio = 2.2; 95% confidence interval = 1.1–4.5).

The onset of cardiovascular adverse events required withdrawal of the TKI treatment in 3.5% of patients treated with nilotinib and 4.3% with ponatinib (Table 2). A reduction of dosing was adopted in 3.1% and 3.2%, respectively. The remaining patients were unchanged in the treatment schedule. Among patients with cardiovascular adverse events, most of them required additional cardiovascular diagnostic tests (coronarography, radiologic imaging, Doppler ultrasound); five required invasive procedures such as percutaneous transluminal angioplasty, bypass, and coronary stent application. Additional medical therapy was introduced in most cases.

## Discussion

Nilotinib and ponatinib effectively treat CML but are potentially associated with cardiovascular complications. Growing evidence suggests that elderly CML patients, more prone to cardiovascular adverse events, may be at higher risk due to the cardiac toxicity associated with using 2ndG/3rdG TKIs. These TKIs have been found to interact with numerous vascular targets that play a crucial role in the survival of endothelial cells and the process of angiogenesis, which are both clinically significant [21]. According to a systematic review and meta-analysis, including ten randomized clinical trials, CML patients treated with nilotinib showed an increased risk of AOEs, with an incidence ranging between 0.5 and 15% after 5 years of observation [18]. Patients who received nilotinib treatment may have a higher likelihood of experiencing a heart attack than those treated with imatinib [22]. Severe adverse cardiovascular effects associated with nilotinib limit its long-term clinical application. The specific causes of these harmful effects are not yet fully understood [23]. Significant cardiovascular toxicity is a challenge for the clinical use of the 3rdG TKI ponatinib. Thus, developing strategies to minimize its toxicity and side effects is necessary [24]. The PACE trial reported a cumulative 5-year incidence rate of AOEs of 31% (serious AOEs, 26%) in the chronic-phase CML population; there was a correlation between a longer duration of treatment with ponatinib and a higher cumulative incidence rate. The occurrence of AOEs was significantly connected to basal cardiovascular risk factors and a previous history of ischemic disease. It is noteworthy that even after a recommended reduction in dosage, the percentage of patients experiencing their first AOE remained the same between those who underwent the dose reduction and those who continued treatment at the same dosage [6]. A review and analysis of three clinical trials found that factors such as dose intensity, history of ischemic disease, and age were the most significant predictors of an increased risk for AOE. This analysis predicted that decreasing the average ponatinib dose intensity by 15 mg/day could lead to a reduction of approximately 33% in the risk of AOE [7].

Thus, these observations suggest that it would be beneficial to tailor treatment plans to each patient, considering their specific cardiovascular risk factors [25]. This could involve selecting the most appropriate TKI for their individual needs and a specific algorithm capable of predicting the cardiovascular risk of each patient.

Recently, the ESC has updated the SCORE algorithm to estimate a 10-year risk of fatal and non-fatal cardiovascular diseases, according to four specific European regions, based on standardized reported WHO age- and sex-standardized overall cardiovascular mortality rates per 100,000 population. The new SCORE2 has been validated on over 1.1 million individuals recruited in the last two decades and 43,000 cardiovascular adverse events [16]. In addition, a specific SCORE2 for older persons (SCORE2-OP) was developed and validated for individuals over 70 years, and this model was calibrated to the same four different geographical European risk regions [17]. Differently from the older SCORE, the SCORE2 and SCORE2-OP estimate not only a 10-year risk of fatal cardiovascular diseases but also a non-fatal risk and are not applicable to patients with a previous history of cardiovascular disease or diabetes because they show a known high risk of cardiovascular complications and specific risk scores already exist for this population. The SCORE2/SCORE2-OP was adjusted on age- and sex-specific relative risks calculated on over 680,000 individuals and included classic cardiovascular risk factors such as sex, age, smoking, systolic pressure, and, interestingly, the variable non-HDL-C instead of total cholesterol.

Non-HDL-C is an estimate of the total amount of pro-atherogenic apolipoprotein B (ApoB)–containing lipoproteins, including very low– and low-density lipoproteins (LDL) [26]. Numerous studies have shown that non-HDL-C is significantly associated with an elevated risk of atherosclerotic cardiovascular disease [27]. In previous studies, low LDL plasma levels were associated with a substantially lower risk of AOEs in CML patients treated with nilotinib and ponatinib in the real life [28, 29]. Dyslipidemia is considered a significant risk factor for cardiovascular disease, and it is becoming more evident that lipoproteins play a crucial role in initiating events in atherogenesis. Atherogenic plaques can form when small ApoB-containing lipoproteins deposit within the arterial wall. This creates a complex inflammatory response that results in the accumulation of lipids [30]. The 2019 guidelines from the ESC emphasize the significance of lipid adjustments in reducing the likelihood of cardiovascular incidents [31]. The experts suggest maintaining cholesterol and triglyceride levels below 200 mg/dL. They also advise following a treatment plan that results in at least a 50% reduction in LDL from the starting point to reach LDL levels lower than 70 mg/dL for patients at high risk of cardiovascular disease and less than 55 mg/dL for those at very high risk [31].

We evaluated the capacity of the SCORE2 and SCORE2-OP to estimate AOEs in a large cohort of 455 consecutive CML patients managed in Italy and treated with nilotinib or ponatinib. Within the four European regions stratified on the standardized cardiovascular disease mortality risk (low, moderate, high, and very high risk), Italy belongs to the moderate-risk region. We found a 20-year cumulative incidence rate of AOEs of 65%, and no difference was found between nilotinib and ponatinib treatments (Fig. 2A, B).

We applied the SCORE2/SCORE2-OP to 380 CML patients without previous cardiovascular diseases or diabetes. The SCORE2 and SCORE2-OP stratified more CML patients at a higher cardiovascular risk than the SCORE. The reason is likely secondary to a recalibration of the age- and sex-adjusted risk, based on the European area of cardiovascular risk, and to consider the variable non-HDL-C instead of total cholesterol (Fig. 1).

We showed that the SCORE2 risk chart had significant predictive value for patients receiving nilotinib and ponatinib treatments; patients who reported a high to very high SCORE2/SCORE2-OP risk showed a notably higher incidence of AOEs (69.2% vs. 46.5%, *p* < 0.001). The SCORE2/SCORE2-OP was able to identify more patients at high to very high risk (Fig. 1) and showed a better specificity in predicting AOEs than the SCORE in patients at low risk (46.5% vs. 54.2%) (Fig. 3A, B).

The SCORE2/SCORE2-OP remained significant in multivariate analysis (*p* = 0.028; Supplemental Table 2). No other significant associations were found between AOE incidence and the older SCORE, gender, age, and nilotinib or ponatinib treatment.

Nilotinib and ponatinib confirmed their efficacy in the CML treatment, obtaining at least MR3 74.6 and 46.7%, respectively. However, in patients developing cardiovascular adverse events, the TKI dose was reduced or interrupted in over half of them. This represents a crucial reason because personalized strategies to minimize the risk of AOEs should be thoroughly screened; this is particularly important for the CML elderly patients that are now included in the SCORE2-OP evaluation. In addition, patients with AOEs require in most cases imaging diagnostic tests, additional drugs, and sometimes invasive procedures, increasing access to visits and hospital management.

Ideally, these patients require the availability of a cardio-oncology facility, being cardio-oncology a discipline based on the collaboration between cardiologists, hematologists, and other medical specialists to prevent, monitor, diagnose, and treat AOEs before, during, and after treatment.

For most patients with chronic myeloid leukemia (CML), long-term treatment with TKI is necessary for survival. Nowadays, these patients can expect a similar survival rate to that of the general population [1]. Therefore, a personalized treatment plan that focuses on disease-free survival and considers quality of life and safety is necessary.

In conclusion, SCORE2 and SCORE2-OP represent helpful charts to estimate a possible risk of AOEs in CML patients treated with nilotinib or ponatinib in real life. They should be presented as a valuable tool in the real-life management of such patients, helping to identify cardiovascular fragility and providing patients with more attention and a proper TKI selection.

### Supplementary Information

Below is the link to the electronic supplementary material.

Supplementary file1 (DOCX 112 KB)

## Data Availability

Database is available at Hematology Unit, Businco Hospital, ARNAS Brotzu Cagliari, Cagliari, Italy.
