# Supplementary material for: The new Systematic Coronary Risk Evaluation (SCORE2 and SCORE2-OP) estimates the risk of arterial occlusive events in chronic myeloid leukemia patients treated with nilotinib or ponatinib
Source: Ann Hematol. 2023 Nov 28;103(2):427–36. doi: 10.1007/s00277-023-05556-0 (PMC10798925; doi:10.1007/s00277-023-05556-0)
Supplement: Supplementary file 1 — Supplementary file1 (DOCX 112 KB) [file 277_2023_5556_MOESM1_ESM.docx]

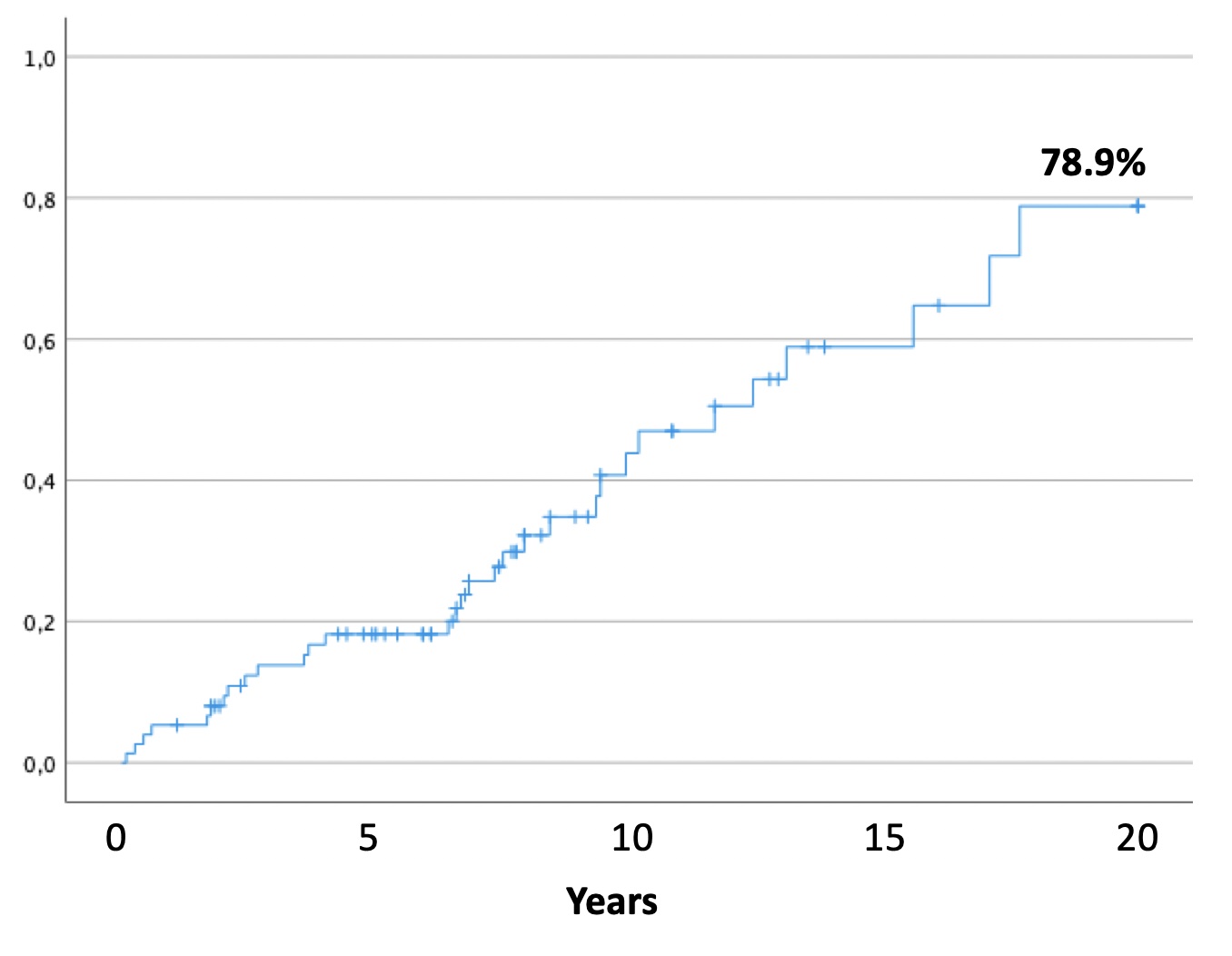


**Supplemental Figure 1. 20-year arterial occlusive events cumulative incidence of 75 CML patients not included in the SCORE/SCORE2/SCORE2-OP classification.**

78.9±9.5% (mean 11.8 years; 95%CI= 10-13.7)

|  | **Nilotinib N= 48** | **Ponatinib N= 27** | **Total N=75** |
| --- | --- | --- | --- |
| *Number of CVEs*, *N (%)* | 21 (43.7) | 11(40) | 32 (42.6) |
| *Number of AOEs*, *N (%)* | 13 (27) | 6 (22.2) | 19 (25.3) |

**Supplemental Table 1. Cardiovascular profile of 75 CML patients not included in the SCORE/SCORE2/SCORE2-OP classification.**

CVEs, cardiovascular events; AOEs, arterial occlusive events

|  | Sign.  univariate | Sign.  multivariate | Exp(B) | 95% C.I. EXP(B) | |
| --- | --- | --- | --- | --- | --- |
|  |  |  |  | Inferior | Superior |
| Sex Male  Age above 60 years  SCORE2/SCORE2-OP high-very high  SCORE high-very high  Nilotinib vs ponatinib | ,244 | ,319 | 1,318 | ,766 | 2,268 |
|  | ,005 | ,314 | 1,395 | ,729 | 2,669 |
|  | <,001 | ,028 | 2,215 | 1,089 | 4,507 |
|  | ,004 | ,397 | 1,308 | ,702 | 2,437 |
|  | ,168 | ,164 | 1,521 | ,843 | 2,743 |

**Supplemental Table 2. Regression logistic multivariate analysis on 380 patients included in the SCORE/SCORE2/SCORE2-OP classification.**

Only the SCORE2/SCORE2-OP variable remained significantly associated with arterial occlusive events.

(HR=2.2; 95%CI 1.1-4.5; p=0.028)
